# Transmembrane Facilitation of Lactate/H^+^ Instead of Lactic Acid Is Not a Question of Semantics but of Cell Viability

**DOI:** 10.3390/membranes10090236

**Published:** 2020-09-15

**Authors:** Annika Bader, Eric Beitz

**Affiliations:** Pharmaceutical and Medicinal Chemistry, Christian-Albrechts-University Kiel, 24118 Kiel, Germany; abader@pharmazie.uni-kiel.de

**Keywords:** lactic acid, lactate, proton, transport, energy metabolism, aquaporin, formate–nitrite transporter, monocarboxylate transporter

## Abstract

Transmembrane transport of monocarboxylates is conferred by structurally diverse membrane proteins. Here, we describe the pH dependence of lactic acid/lactate facilitation of an aquaporin (AQP9), a monocarboxylate transporter (MCT1, SLC16A1), and a formate–nitrite transporter (plasmodium falciparum FNT, PfFNT) in the equilibrium transport state. FNTs exhibit a channel-like structure mimicking the aquaporin-fold, yet act as secondary active transporters. We used radiolabeled lactate to monitor uptake via yeast-expressed AQP9, MCT1, and PfFNT for long enough time periods to reach the equilibrium state in which import and export rates are balanced. We confirmed that AQP9 behaved perfectly equilibrative for lactic acid, i.e., the neutral lactic acid molecule enters and passes the channel. MCT1, in turn, actively used the transmembrane proton gradient and acted as a lactate/H^+^ co-transporter. PfFNT behaved highly similar to the MCT in terms of transport properties, although it does not adhere to the classical alternating access transporter model. Instead, the FNT appears to use the proton gradient to neutralize the lactate anion in the protein’s vestibule to generate lactic acid in a place that traverses the central hydrophobic transport path. In conclusion, we propose to include FNT-type proteins into a more generalized, function-based transporter definition.

## 1. Introduction

**Figure 1:**
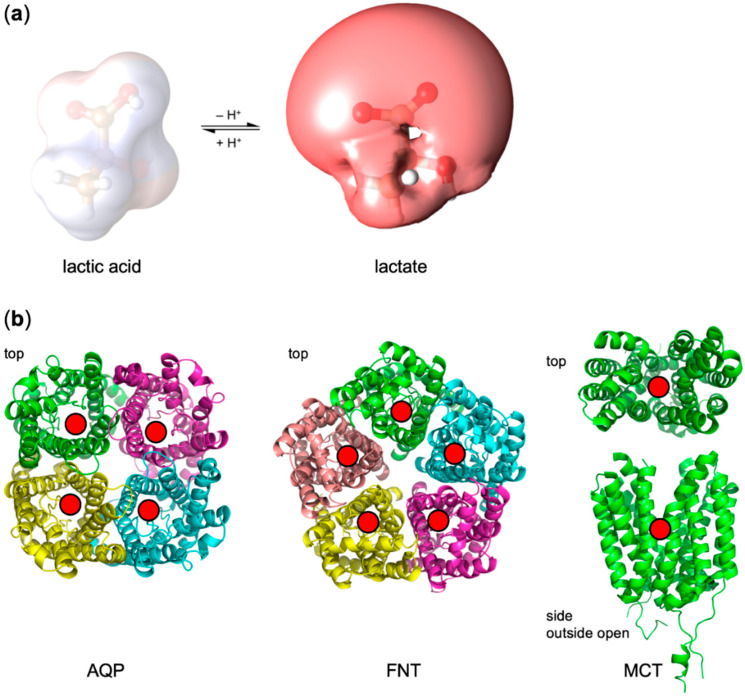
(**a**) Lactic acid/lactate protonation equilibrium. Shown are the molecule structures and electrostatic potential maps (blue/red scale: ± 4 kT e^–^). (**b**) Structures of membrane proteins facilitating transmembrane lactate transport. Aquaporin homotetramers (AQP; PDB# 1FX8) harbor one substrate (red circle) channel in each protomer. Formate–nitrite transporters (FNT; PDB# 3KCU) exhibit a structure mimicry with the AQPs on the protomer level, yet function like proton-driven transporters. Alternating access monocarboxylate transporters (MCT; PDB# 6HCL) bind the substrate and a proton on one side and undergo a conformational change for release at the trans-side of the membrane.

Weak acids (lactate, pyruvate, acidic ketone bodies, etc.) are fundamental in energy metabolism throughout evolution and all kingdoms of life. Their transmembrane transport enables, for instance, ancient bacterial mixed acid fermentation in oxygen-free atmospheres [1] or the modern Krebs cycle by shuttling pyruvate into mitochondria [2], fuels the human brain with ketone bodies during glucose limitation [3] or via the astrocyte-neuron lactate shuttle [4], promotes tumor growth by lactate release (Warburg effect) [5,6], or lets malaria parasites thrive by a similar mechanism [7]. Because of the high polarity of weak acid molecules and the full negative charge of the corresponding anion, diffusion across the lipid bilayer is marginal. Instead, a variety of integral membrane proteins facilitate the transmembrane passage [1,2,3,4,5,6,7]. During transport, weak acids can act as true shape-shifters (Figure 1a). Contrary to permanently charged (e.g., choline) or uncharged substrates (e.g., glycerol, urea), weak acids undergo protonation/deprotonation events at physiological pH, and, likely, also act as proton acceptors and donors within the protein context [8,9]. It is possible that their protonation status is actively changed when traversing a protein’s transport path for selectivity purposes. Changing a weak acid substrate’s character during transport should have major effects as charged and neutral molecules behave fundamentally different regarding affinity (electrostatic vs. van der Waals interactions), the size of hydration shell and dehydration penalty, impact on membrane potentials (ions) or absence thereof (neutrals), and dependence on the environmental pH (proton co-transport).

In this study, we compared the dependence on the transmembrane pH gradient of three different types of lactate-conducting proteins. These are a strict neutral solute channel (human aquaporin 9, AQP9) [10], a prototypical secondary active, proton-driven alternating access monocarboxylate transporter (human MCT1; SLC16 family) [11,12], and the malaria parasite’s lactate transporter (PfFNT; microbial formate–nitrite transporter family) [7,13] (Figure 1b). The latter represents an in-between case: although the FNTs form homopentamers, their rigid protomer structure mimics the fold of an aquaporin (AQP) channel [14], whereas the lactate facilitation mechanism appears proton driven, i.e., secondary active transporter-like [8,9]. At the same time, the substrate affinity of the FNTs is in the double-digit millimolar range, i.e., channel-like low [7,8]. The FNT central transport path is shielded on either side by a constriction that is too narrow and too hydrophobic for a monocarboxylate anion to pass [15,16,17]. To overcome this barrier, the substrate is neutralized by protonation in the vestibule leading to the constriction, and subsequently the amino acid sidechains in the constriction region give way while the backbone remains in place [1]. The classical definition of a transport protein demands that only one entry site is open at a time for the binding of substrate [18]. This requirement is not met by the FNTs, bringing up the question whether FNTs should not be classified as transporters or whether the definition needs to be adapted.

## 2. Materials and Methods

### 2.1. Expression Plasmids, Yeast Transformation and Culture

Yeast expression constructs of human AQP9 and codon-optimized PfFNT in pDR196 (Addgene #36029) have been described before [7,10]. DNA encoding the open reading frame of human MCT1 (GenBank NM_001166496) in pcDNA3.1+/C-(K)DYK was obtained commercially (GenScript, Leiden, Netherlands), and cloned into pDR196 using PflMI and BspEI restriction sites. Respective restriction sites were generated by PCR. All constructs were designed to carry an N-terminal hemagglutin epitope (HA-tag) and a C-terminal His_10_-tag, and checked by sequencing. The *Saccharomyces cerevisiae* yeast strain W303-1A jen1Δ ady2Δ (MATa, can1-100, ade2loc, his3-11-15, leu2-3,-112, trp1-1-1, ura3-1, jen1::kanMX4, ady2::hphMX4) lacking endogenous monocarboxylate transporters was kindly provided by M. Casal [19]. Transformation was done using the lithium acetate/single stranded carrier DNA/polyethylene glycol method [20]. Transformed cells were grown at 29 °C in selective medium (synthetic drop-out, SD) complemented with adenine, histidine, leucine and tryptophan, and 2% (*w*/*v*) glucose, without uracil. For the phenotypical assay of yeast cell growth, 2% l-lactate was used instead of glucose. The agar media were buffered to pH 5.8 and pH 6.8, respectively, with 50 mM MES.

### 2.2. Isolation of Membrane Proteins and Western Blot

A total of 40 mL of a yeast cell culture was grown to an OD_600_ of 1 and harvested by centrifugation (4000× *g*, 5 min). Cells were washed with 25 mL water and 10 mL extraction buffer (5 mM EDTA, 25 mM Tris, pH 7.5) and resuspended in 0.5 mL extraction buffer and stored at −80 °C for at least 30 min. Cell disruption was done by adding 0.5 g acid-washed glass beads (Sigma-Aldrich, Darmstadt, Germany, Ø 425–600 µm) and 15 cycles of vortexing. Between each cycle, cells were stored on ice for 30 s. The supernatant was cleared at 10,000× *g*, 4 °C for 5 min and the membrane fraction was collected at 100,000× *g*, 4 °C for 40 min. The pellet was resuspended in 50 µL of 100 mM phosphate buffer, pH 8.0 and 50 mM NaCl. The total protein amount was determined using the Bradford Protein Assay (Bio-Rad, Feldkirchen, Germany). A total of 5–10 µg of total protein was separated via SDS-PAGE and blotted on polyvinylidene fluoride (PVDF) membranes (Hybond P 0.45, GE Healthcare, München, Germany). The probes were detected with a monoclonal mouse anti-HA antibody at 1:5000 dilution (12CA5, Roche; cat. no. 11583816001), horseradish peroxidase conjugated secondary goat-anti-mouse antibody at 1:5000 dilution (Jackson ImmunoResearch/Dianova, Hamburg, Germany; cat. no. 115-035-174) and the Clarity ECL substrate detection system (Bio-Rad, Feldkirchen, Germany). Imaging was done with the Chemostar Touch ECL and Fluorescence Imager (Intas Science Imaging Instruments, Göttingen, Germany).

### 2.3. l-lactate Uptake Assay

A previously established protocol was used [7]. Briefly, yeast cells were grown overnight to an OD_600_ of 1.0 ± 0.1, harvested by centrifugation at 4000× *g*, 4 °C for 5 min, washed once with ice-cold water, and resuspended to an OD_600_ of 50 ± 5 in the buffer of interest: 50 mM HEPES/Tris (pH 6.8), 50 mM MES/Tris (pH 5.8), 50 mM citric acid/Tris (pH 4.8 and pH 3.8). For radiolabeled l-lactate uptake, 20 µL of 5 mM l-lactate spiked with 0.04 µCi of ^14^C-labeled l-lactate (Hartmann Analytics, Braunschweig, Germany) were added to 80 µL of cell suspension in a 1.5 mL tube to yield a final concentration of 1 mM l-lactate. The reaction was stopped by adding 1 mL of ice-cold water, transferring the suspension to a GF/C glass microfiber filter membrane (Whatman/Sigma-Aldrich, Darmstadt, Germany) and removing the supernatant by vacuum filtration. The filters were washed with 7 mL of ice-cold water and transferred to scintillation vials containing 3 mL of scintillation cocktail (Quicksafe A; Zinsser Analytic) for counting (Packard TriCarb liquid scintillation counter, Perkin Elmer Inc., Waltham, MA, USA). Single-exponential (uptake data) and sigmoidal fittings (pH-dependent capacity) were calculated after subtraction of the background from non-expressing yeast cells (SigmaPlot version 11.0). Measurements were done in triplicate (independent yeast cultures) with three technical replicates each, and error bars denote the standard error of the mean (SEM).

### 2.4. Determination of the Cytosolic pH in Yeast

The protocol was adapted from Bracy et al. [21]. Calibration was done using 30 μg·mL^−1^ of 5′(6′)-carboxyfluoresceindiacetatesuccinimidylester (CFDA-SE, Sigma Aldrich, Darmstadt, Germany) cleaved overnight by 50 μg mL^−1^ porcine esterase (Sigma Aldrich, Darmstadt, Germany) in 1 mL of water at room temperature. The cleaved probe was added to 50 mM citric/phosphate buffers with varying pH from 3.8 to 6.8 in 0.2 pH steps. The calibration was replicated three times, once with and twice without permeabilized yeast cells (60 min at 30 °C with 4.37 µM amphotericin (Sigma Aldrich, Darmstadt, Germany)), and pH-dependent fluorescence intensity ratios (λ_em_ = 525 nm) were determined from λ_ex_ = 495 (pH-dependent) and λ_ex_ = 435 nm (isobestic point). The data were averaged from all three replicates and the resulting pH curve was plotted using the polynomial function f(x) = −3.4238 + 5.4077 x − 1.9197 x^2^ + 0.2033 x^3^ (SigmaPlot). The error bars represent SEM.

The intracellular pH of yeast was measured with cells carrying the empty pDR196 plasmid. Cells (OD_600_ of 10) were loaded at 37 °C for 24 h with 100 µM CFDA-SE in 50 mM citric/phosphate buffer (pH 4.0), collected and washed before resuspension in the pH buffer of interest. The cells were kept at these conditions for at least 30 min for equilibration [21]. Determinations were done in triplicate for each buffer pH of 3.8, 4.8, 5.8 and 6.8; prior to measurements, the cell suspensions were diluted to match the intensity range of the fluorometer (LS 55 using a QS 4/4 mm cuvette PerkinElmer, Waltham, MA, USA). To minimize background, each cell suspension was subsequently cleared by centrifugation (10,000× *g*, 5 min) and the fluorescence signal of the supernatant was subtracted. The error bars denote SEM.

## 3. Results

**Figure 2:**
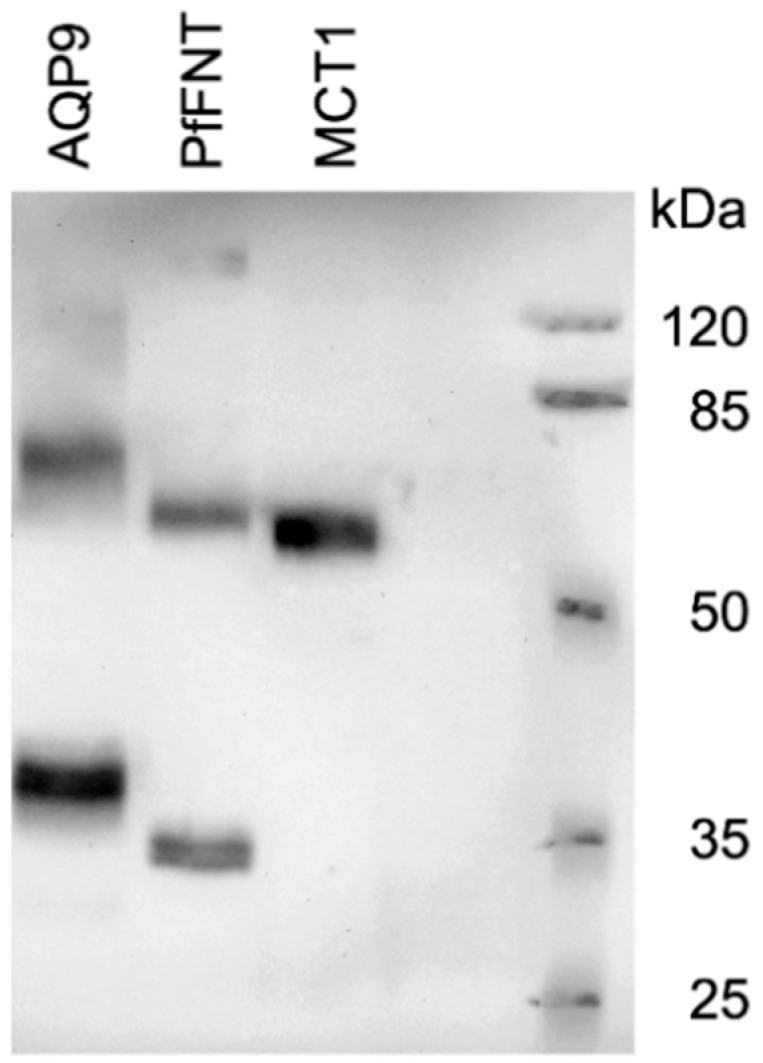
Western blot showing AQP9 (39 kDa monomer molecular weight), PfFNT (plasmodium falciparum FNT, 34 kDa monomer), and MCT1 (60 kDa) in yeast.

We produced AQP9, MCT1, and PfFNT in a *S. cerevisiae* yeast knockout strain (W303-1A *jen1Δ ady2Δ*) that lacks endogenous monocarboxylate transporters [19]. All proteins expressed well and gave the typical appearance in the Western blot (Figure 2) [7,10,13]. MCT1 yielded a single signal at the expected molecular weight, whereas AQP9 and PfFNT showed prominent monomer and dimer bands as well as higher order complexes of weaker intensity.

In the following, we exposed expressing yeast suspensions to 1 mM inward gradients of radiolabeled external lactate and followed the uptake over time. The use of a yeast monocarboxylate transporter knockout yeast strain and the glucose-rich culture conditions allowed us to measure uptake for prolonged times of 15 min without interference of background transport or metabolic conversion of lactate [22]. During this time period, the assays typically reached the transport equilibrium state (or could be extrapolated), K_eq_, at which import and export rates (k_inward_, k_outward_) are balanced and a certain intracellular/extracellular lactate concentration ratio (c_in_, c_ex_) has established: K_eq_ = k_inward_/k_outward_ = c_in_/c_ex_ [23]. This ratio is termed uptake capacity and is pH dependent because of the protonation equilibrium of the substrate. The uptake capacity is further affected by the directionality of the transport protein. AQP9 as a neutral solute channel [24], however, is equilibrative for the uncharged lactic acid. We set out to use this system to assess the pH dependent lactate uptake capacity of PfFNT as a parameter that should characterize its transport mechanism as either channel- or transporter-like.

### 3.1. AQP9 Facilitates Uptake of Neutral Lactic Acid Following the Substrate Protonation Equilibrium

**Figure 3:**
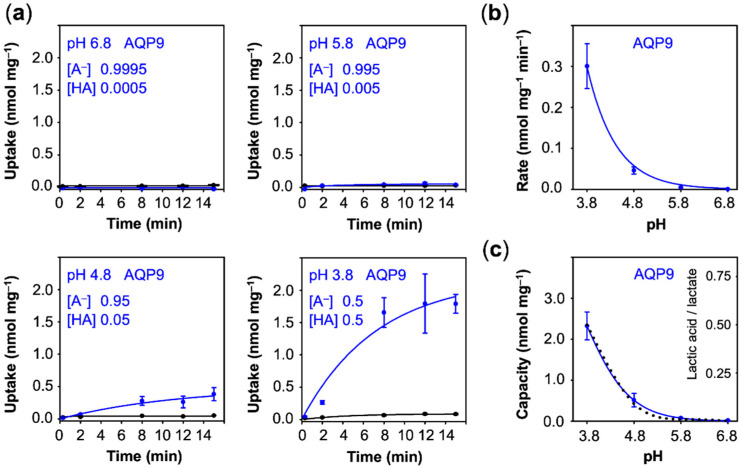
pH-dependent uptake of lactate/lactic acid into AQP9 expressing yeast. (**a**) Shown are uptake curves over time using cells with AQP9 (blue) compared to non-expressing cells (black). The proportion of external lactate anions [A^–^] and neutral lactic acid [HA] is indicated for each pH condition. (**b**) Uptake rates derived from the uptake curves. (**c**) Uptake capacity obtained from the plateaus of the uptake curves. The dotted line indicates the proportion of neutral lactic acid at a given pH condition. Error bars indicate the standard error of the mean (SEM) from three independent experiments.

First, we monitored the lactate/lactic acid uptake of yeast cells expressing AQP9 adjusted to acidic buffer conditions for at least 30 min prior to the experiment. The most acidic pH of 3.8 corresponds to the pK_a_ of the lactate substrate, i.e., the concentration of charged lactate and neutral lactic acid was even at 0.5 mM, whereas as at pH 6.8, 0.9995 mM lactate and only 0.0005 mM of neutral lactic acid were present in the buffer. At close to neutral pH 6.8 and at pH 5.8, we did not observe uptake above the background level (Figure 3a). Uptake became measurable at an external pH of 4.8 (rate: 0.05 nmol·mg^−1^·min^−1^) and increased by one order of magnitude when further shifted to pH 3.8 (rate: 0.30 nmol·mg^−1^·min^−1^), while background membrane diffusion of the neutral lactic acid increase only marginally (Figure 3a,b). Since the AQP9 channel strictly excludes the passage of charged substrates [25], the observed increase in uptake is due to an increased proportion of protonated, neutral lactic acid at more acidic pH conditions. The total load of lactate/lactic acid of the yeast cells equally increased with the acidity of the external buffer and perfectly followed the proportion of neutral lactic acid that is present at the respective pH condition (Figure 3c, dotted line).

### 3.2. MCT1 Transports Lactate at Neutral pH and Actively Uses the Transmembrane Proton Gradient

**Figure 4:**
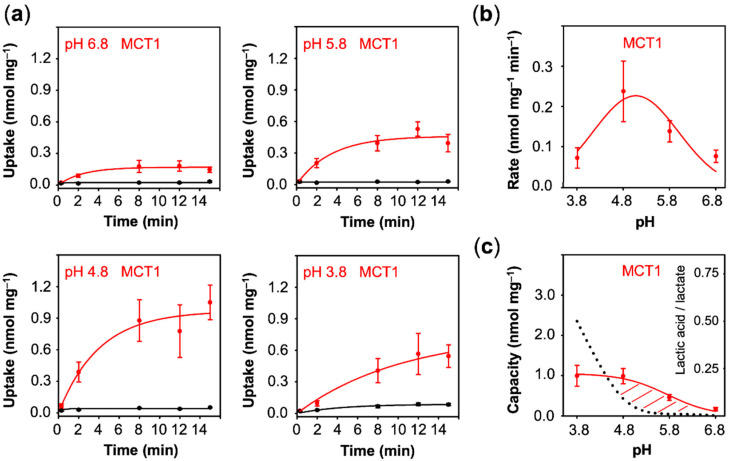
pH-dependent uptake of lactate/lactic acid into MCT1 expressing yeast. (**a**) Shown are uptake curves over time using cells with MCT1 (red) compared to non-expressing cells (black). (**b**) Uptake rates derived from the uptake curves. (**c**) Uptake capacity obtained from the plateaus of the uptake curves. The dotted line indicates the proportion of neutral lactic acid at a given pH condition. Error bars indicate SEM from three independent experiments.

We next assayed lactate uptake of cells expressing MCT1. Clearly, MCT1 transported lactate even at neutral pH conditions (Figure 4a). Acidification further increased the uptake up to pH 4.8 (0.24 nmol·mg^−1^·min^−1^). The equilibrium state was reached already around 8 min, indicating that, contrary to the aquaporin channel, the transmembrane proton gradient is actively used by the transporter to facilitate lactate uptake. At the lowest, non-physiological pH of 3.8, the uptake decreased again (0.07 nmol·mg^−1^·min^−1^) (Figure 4b), yet extrapolation suggested a similar capacity to that at pH 4.8 (1.00 nmol·mg^−1^) (Figure 4c). The decline in the transport rate at the lowest pH may be due to the disturbance of the MCT1 protein by protonation, e.g., at Asp, Glu, or His amino acid sidechains, or to the 47% decrease in the proportion of the lactate anion from 0.95 mM at pH 4.8 to 0.5 mM at pH 3.8, or a combination of both.

### 3.3. Lactate Uptake via PfFNT is Secondary Active Transporter-Like

**Figure 5:**
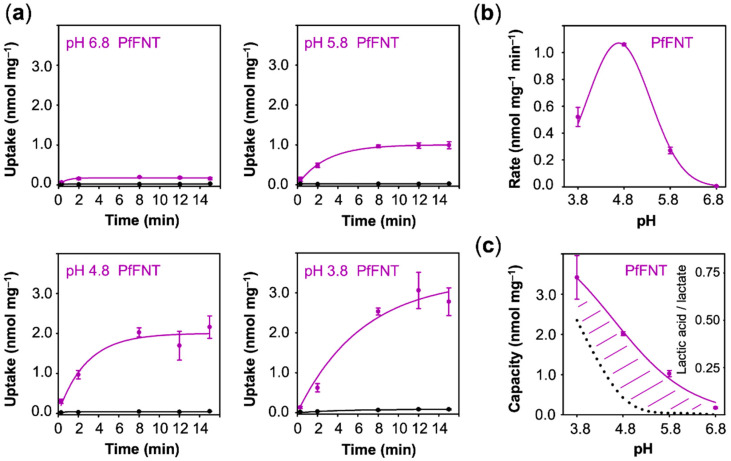
pH-dependent uptake of lactate/lactic acid into PfFNT expressing yeast. (**a**) Shown are uptake curves over time using cells with PfFNT (purple) compared to non-expressing cells (black). (**b**) Uptake rates derived from the uptake curves. (**c**) Uptake capacity obtained from the plateaus of the uptake curves. The dotted line indicates the proportion of neutral lactic acid at a given pH condition. Error bars indicate SEM from three independent experiments.

The different behavior of AQP9 and MCT1 regarding the pH dependence of lactate/lactic acid transport was expected because earlier structural and functional studies classified the proteins as channel [24] and secondary active transporter [26], respectively. The case was less clear for PfFNT, which, structure wise, resembles an aquaporin channel [14], whereas previous functional investigations revealed transporter-like properties [7,8,9]. Our assays with PfFNT showed some uptake already at pH 6.8 (Figure 5a). Similar to MCT1, the uptake rates increased with the external acidity up to a pH of 4.8 (1.10 nmol·mg^−1^·min^−1^) and then declined (0.52 nmol·mg^−1^·min^−1^) (Figure 5b). The curve representing pH dependence of the uptake capacity also appeared similar in shape to that of MCT1 (Figure 5c).

Contrary to uptake rates, capacity values derived from equilibrium transport conditions are independent from the actual number of transport proteins at the plasma membrane. With fewer transporters, the plateau would be reached after longer times, yet the absolute value would not change. This allows one to compare transport properties of different proteins of probably different expression levels. The capacity further mirrors the ratio of the rate constants of substrate import and export. It is striking that the AQP9 capacity curve perfectly followed the availability of neutral lactic acid in the external buffer, i.e., it is equilibrative for lactic acid (dashed line in Figure 3c), whereas MCT1 and PfFNT confer intracellular substrate loads greater than the external lactic acid proportion (positive deviations from the dotted lines in Figure 4c and Figure 5c). Together with the observed proton-driven transport, this suggests that MCT1 and PfFNT attract lactate anions and act as co-transporters for lactate anions and protons. This is a clear indication that PfFNT, despite exhibiting an aquaporin-like fold, has secondary active transporter-like properties.

### 3.4. Relating Transport to the True Transmembrane Proton Gradient and Physiological Relevance

**Figure 6:**
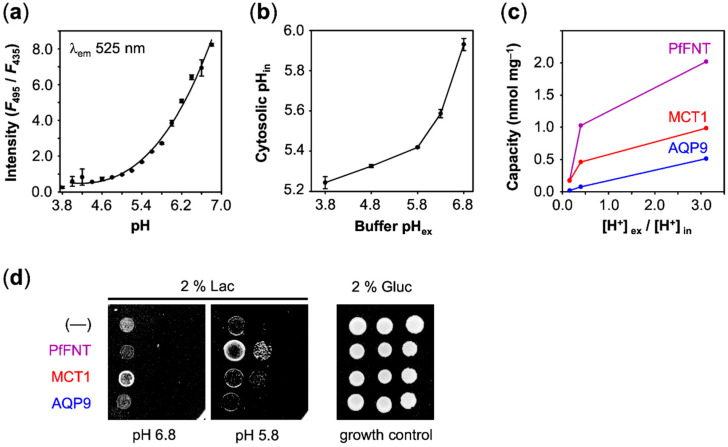
Determination of true transmembrane proton gradients and effect of lactate uptake on yeast growth. (**a**) Calibration of the fluorescence intensity ratio to pH. (**b**) Determination of the yeast cytosolic pH adaptation in response to the external buffer pH. (**c**) Dependence of the lactate/lactic acid uptake capacity on the true transmembrane proton gradient (pH range 6.8 to 4.8). (**d**) Lactate dependent yeast growth (1:10 serial dilutions on agar plates) facilitated by PfFNT, MCT1, and AQP9.

The above uptake measurements were carried out using defined extracellular buffer pH conditions, yet without knowledge of the cytosolic pH. However, the ratio of the external and internal proton concentration, [H^+^]_ex_/[H^+^]_in_, defines the true transmembrane gradient. To determine the cytosolic yeast pH, we employed a fluorescence method based on pH sensitive carboxyfluorescein [21]. The probe can be used in the range from pH 4 to 7 to determine the fluorescence intensity ratio at λ_em_ = 525 nm after excitation, at λ_ex_ = 495 nm and λ_ex_ = 435 nm (Figure 6a). When applied to yeast cells, we generally found a slightly acidic cytosolic pH, which is probably due to adaptation to the acidic culture conditions. When we shifted the cells from the growth media (pH 5.6) to an external buffer of pH 6.8, we still determined a cytosolic pH of 6.0 (Figure 6b). The cytosolic pH dropped to pH 5.2 with increasing acidity of the external buffer. The resulting [H^+^]_ex_/[H^+^]_in_ ratios that we derived from the measurements are shown in Table 1.

Eventually, we plotted the capacity values obtained with AQP9, MCT1, and PfFNT (Figure 3c, Figure 4c and Figure 5c) against the true transmembrane proton gradient (Figure 6c). In this display, AQP9 linearly followed the transmembrane gradient indicating uptake of neutral lactic acid strictly according to the chemical protonation equilibrium. MCT1 and PfFNT, in turn, exhibited enhanced uptake even at low external proton concentrations, which is typical for proton-driven, secondary active transport.

Finally, we asked whether cell viability depended on the observed lactate-facilitating properties of AQP9, PfFNT, and MCT1. Therefore, we monitored the growth of yeast on agar media containing lactate as the sole carbon source to test which protein is capable of facilitating physiologically meaningful transmembrane lactate uptake. Just as the background control (––), AQP9 was unable to sustain lactate dependent growth in the tested pH range (Figure 6d). At the most neutral pH 6.8 condition, only MCT1 promoted cell growth above the background. The observed higher transport capacity of PfFNT at pH 5.8 over MCT1 (see Figure 6c) accordingly resulted in increased yeast growth with PfFNT under these pH conditions (Figure 6d).

## 4. Discussion

AQP9, PfFNT, and MCT1 facilitate a net transport of electroneutral lactic acid across the plasma membrane. The acidity of lactic acid though being relatively weak (pK_a_ 3.8) still leads to strong dissociation at buffered physiological pH conditions, leaving undissociated lactic acid concentrations in the per mille range: log([HA]/[A^–^]) = pK_a_ − pH (Henderson–Hasselbalch) [27,28]. Therefore, proteins that take neutral lactic acid as a transport substrate act rather inefficiently due to the low concentration of available substrate.

**Figure 7:**
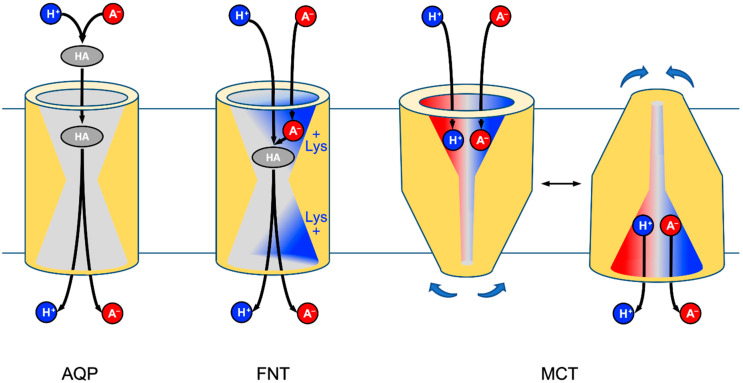
Substrate acquisition and transmembrane transfer at the protomer level of AQPs (neutral solutes), FNTs (substrate anion attraction and protonation due to dielectric environment), and MCTs (alternating access secondary active co-transport of substrate and proton).

AQP9 represents the class of neutral solute facilitators with a simple substrate selection mechanism by size-exclusion (Figure 7, left) [29,30]. The AQP channel interior excludes the passage of charged molecules and the protein lacks mechanisms to compensate for the energetic cost of stripping off a charged substrate’s hydration shell [31,32]. As a result, acidic pH conditions typically below the physiological range are required to increase the proportion of free lactic acid and to promote transmembrane permeation. AQP9 in particular as well as certain aquaporins, e.g., from *Lactobacillus* species carry clusters of positively charged amino acid residues at the protein surface, that appear to attract anions incl. lactate and, thus, slightly increase the concomitant local concentration of lactic acid [10,33].

In order to be efficient at physiological, i.e., low proton, pH conditions, an apparent solution for a channel/transporter would be to take the predominant lactate anion as a substrate. However, this would lead to problems in either transport direction. Uptake of lactate anions into the cytosol would be limited to the single-digit percent range by the negative membrane potential: ln([A^−^]_ex_/[A^−^]_in_) = −Δ*ψ F/R T* (Gibbs-Donnan equilibrium; Δ*ψ*, membrane potential; *F*, Faraday constant; *R*, gas constant; *T*, absolute temperature) [34]. The release of lactate anions from the cell, in turn, would leave the protons behind and lead to rapid acidification the cytosolic pH. FNTs and MCTs, thus, have evolved ways to attract lactate anions as well as neutralizing protons for co-transport in the import as well as export direction [7,26].

FNTs are symmetrical with respect to the plane of the membrane and carry a conserved lysine residue in either vestibule leading to a hydrophobic center of the transport path (Figure 7) [35]. We favor a transport model in which lactate anions are electrostatically attracted by the positively charged lysine [8]. Upon entering the vestibule, the substrate encounters an increasingly less polar dielectric environment. At a certain point down the “dielectric slide” [9] a proton, likely from the aqueous bulk solution, will be transferred to the lactate anion for neutralization. The formed neutral lactic acid passes—after an aquaporin-like size-exclusion checkpoint [16]—the hydrophobic protein core and is released at the *trans*-side of the membrane. Previous electrophysiological measurements showed that some monocarboxylate anions pass the FNT when the availability of protons is limited (pH > 6) [1]. MCTs appear more specialized for the task as they bind lactate anions at a dedicated substrate binding site [11] at one order of magnitude higher affinity than FNTs [36,37]. Additionally, the proton co-substrate is bound. However, the actual proton binding site and transfer mechanisms are not known, yet [11]. Subsequently, MCTs undergo a major conformational change (Figure 7, right) that closes the *cis*-side and opens up the *trans*-side, where lactate and the proton are released [11,12].

The major difference in mechanism between the structure-related AQPs and FNTs is the relocation of the lactate-proton-transfer site into the protein environment. This way, the FNTs make use of the much larger pool of lactate, which increases the efficiency of transport by orders of magnitude and renders it physiologically meaningful. Earlier, we identified a similar principle in a natural AQP-enzyme fusion that confers arsenite resistance in certain seawater bacteria [38]. While AQPs act as lactic acid channels, the FNTs functionally represent lactate/H^+^ co-transporters that are comparable in efficiency to the MCTs. Evolutionary, the FNT-type of monocarboxylate transport remained restricted to microbes [39,40,41], whereas the alternating-access mechanism of the MCTs with a more specific substrate binding site certainly constitutes a more modern principle.

In conclusion, with the identification of FNTs that were clearly derived from channel proteins yet adapted secondary active transport properties by generating simple enzymatic reaction chambers in the vestibules, we propose that the term “transporter” should be based on function rather than structure, and the FNTs should be fully endorsed.

## Figures and Tables

**Table 1:** Yeast transmembrane proton gradients at various external pH conditions.

pH_ex_	[H^+^]_ex_ (µM)	pH_in_	[H^+^]_in_ (µM)	[H^+^]_ex_/[H^+^]_in_
6.8	0.16	6.0	1.00	0.16
5.8	1.58	5.4	3.98	0.40
4.8	15.58	5.3	5.01	3.11
